# *De novo* transcriptome assembly of four organs of *Collichthys lucidus* and identification of genes involved in sex determination and reproduction

**DOI:** 10.1371/journal.pone.0230580

**Published:** 2020-03-27

**Authors:** Wei Song, YiBing Zhang, XiaoJuan Zhang, JianFang Gui

**Affiliations:** 1 Institute of Hydrobiology, Chinese Academy of Sciences, Wuhan, China; 2 East China Sea fisheries Research Institute, Chinese Academy of Fishery Sciences, Shanghai, China; Xiamen University, CHINA

## Abstract

The spinyhead croaker (*Collichthys lucidus*) is a commercially important fish species, which is mainly distributed in the coastal regions of China. However, little is known about the molecular regulatory mechanism underlying reproduction in *C*. *lucidus*. A *de novo* transcriptome assembly in brain, liver, ovary and testis tissues of *C*. *lucidus* was performed. Illumina sequencing generated 60,322,004, 57,044,284, 60,867,978 and 57,087,688 clean reads from brain, liver, ovary and testis tissues of *C*. *lucidus*, respectively. Totally, 131,168 unigenes with an average length of 644 bp and an N50 value of 1033 bp were assembled. In addition, 1288 genes were differentially expressed between ovary and testis, including 442 up-regulated and 846 down-regulated in ovary. Functional analysis revealed that the differentially expressed genes between ovary and testis were mainly involved in the function of sexual reproduction, sex differentiation, development of primary male sexual characteristics, female gamete generation, and male sex differentiation. A number of genes which might be involved in the regulation of reproduction and sex determination were found, including *HYAL* and *SYCP3* and *BMP15*. Furthermore, 35,476 simple sequence repeats (SSRs) were identified in this transcriptome dataset, which would contribute to further genetic and mechanism researches. *De novo* transcriptome sequencing analysis of four organs of *C*. *lucidus* provides rich resources for understanding the mechanism of reproductive development of *C*. *lucidus* and further investigation of the molecular regulation of sex determination and reproduction of *C*. *lucidus*.

## Introduction

*Collichthys lucidus*, which belongs to Perciformes, is an economically important fish with small somatotype and usually lives in the benthic zone of coastal waters [1]. It’s widely distributed in the middle and western Pacific Ocean, including Japan, Korea and China [2]. *C*. *lucidus* is appreciated by customers because of its good quality of meat, which contributing to fishery production [3]. Statistics showed that it reached 222.6t per year [4]. In recent years, studies revealed that the resources of *C*. *lucidus* have been declining due to the overfishing and the damage of environment [5]. Therefore, it is necessary to carry out genetic research and exploit reproductive techniques to increase fish production for resource protection and ecological balance.

The hypothalamic-pituitary-gonadal (HPG) axis plays a key role in the regulation of animal reproduction and development through the secretion of reproductive hormones regulation [6, 7]. Gonadotropin-releasing hormone (GnRH) is a key neuroendocrine regulator of the HPG axis, and its m RNA expression level keeps changing during the gonadal development cycle, which is positively correlated with gonadal development [8]. Studied reported that GnRH m RNA expression level was remarkably different during the ovarian development cycle of fish such as scorpion *Sebastes rastrelliger*, and *Oncorhynchus mykiss* [9,10]. Abnormal HPG axis can lead to defective reproductive performance of animals. As previous article reported, a variety of natural or synthetic compounds, such as monocrotophos pesticide, semicarbazide and thyroid hormones, could lead to significant effect on fish reproduction through HPG [11–13]. For example, Hou et al. investigated the effects of norethindrone on gonad differentiation and sex determination of juvenile zebrafish *Dario renio*, and revealed that the expressions of genes associated with the HPG axis was altered by exposure to norethindrone, thus resulting in affecting sex differentiation [14]. In addition, Liang et al. found that progesterone had effects on reproduction by changing the transcriptional expression of genes related to HPG axes during the early development of zebrafish *Dario renio* [15]. However, the regulation of reproduction through this axis in *C*. *lucidus* is still unknown.

In the past few years, studies on *C*. *lucidus* mostly focused on feeding ecology and genetic diversity [16, 17]. However, the molecular mechanisms of *C*. *lucidus* have rarely been studied. Transcriptome sequencing is an effective method to investigate genes involved in specific biological processes and is also a rapid and effective method to discover molecular markers such as SSRs, single nucleotide polymorphisms (SNPs) [18]. With the rapid development of high-throughput sequencing, the transcriptome sequencing have been widely used. Liu et al. performed *de novo* transcriptome sequencing of muscle tissues of *C*. *lucidus* and provided rich molecular resources for *C*. *lucidus* [19]. Chen et al. obtained 78,671 putative SNPs using RNA-seq and demonstrated that 29 SNP loci exhibited bi-allelic polymorphism in genotyping [20]. However, the gonad transcriptome data of *C*. *lucidus* is not currently available.

The HPG axis plays an important role in fish reproductive development. The liver is the main hormone secreting organ in fish, and the development of fish gonads requires the participation and regulation of various steroid hormones. Therefore, in this study, brain, liver, ovary, and testis tissues of *C*. *lucidus* were sequenced and *de novo* assembled to obtain the transcriptome data and discover genes that might be involved in the regulation of reproductive development using Illumina NextSeq 500 platform. The analysis of transcriptome sequences will provide valuable resources for understanding the mechanism governing reproductive development in *C*. *lucidus* and it will be helpful for the further research on reproduction of *C*. *lucidus*. Furthermore, the identification and analysis of SSR loci and the molecular markers obtained in our study will lay the foundation for the research of population genetics in *C*. *lucidus*.

## Materials and methods

### Ethical statement

The collection of samples and the handling of animals in this article were approved by the Chinese Academy of Fishery Sciences Welfare Committee, Shanghai, China. All procedures conducted with the fishes were performed in accordance with relevant guidelines and regulations. All efforts were made to minimize animal suffering.

### Sample collection and RNA extraction

Five wild *C*. *lucidus* individuals were obtained from Ningde sea area, Fujian province, China. Fishes were euthanized by intraperitoneal injection with excessive pentobarbital (150mg/kg). Brain, liver, ovary and testis tissues of *C*. *lucidus* weighing about 0.5 grams each were harvested. All samples were saved in RNAlater (TransGen Biotech, Beijing, China) and then stored at -80°C for RNA extraction. Total RNAs of each sample were extracted using Animal Total RNA Isolation Kit (Foregene, Chengdu, China) following the introductions by manufacture. Total RNA concentration was measured by Nanodrop 2000 (Invitrogen, Carlsbad, CA, USA), and the RNA integrity was evaluated by Agilent 2100 Bioanalyzer (Agilent Technologies, CA, USA).

### cDNA library preparation and sequencing

Qualified RNA was processed using Genomic Sample Preparation kit (Illumina, Beijing, China) for library construction. Briefly, mRNA was isolated using magnetic beads with Oligo (dT) and the mRNA was then mixed with fragmentation buffer to obtain short fragments of 200-300bp. Next, the fragments were used to synthesize first-strand cDNA with random primers, and first-strand cDNA was transformed into double-strand cDNA by using DNA Polymerase I and RNase H. Then these cDNA fragments were processed by an end-repair and the ligation of adapters according to the manufacturer’s protocol (Beckman Coulter, Beverly, USA). The products were purified and enriched with PCR. Finally, the cDNA library qualified with an Agilent Bioanalyzer 2100 system. The resultant cDNA libraries were sequenced on the Illumina NextSeq 500 platform (Illumina, USA).

### Sequence data processing and *de novo* assembly

To obatain high-quality clean reads, raw reads with adaptors and poly-N, and low-quality reads containing more than 50% bases with a Q-values <20 were removed. *De novo* assembly with clean reads was performed using Trinity software, by which transcripts and unigenes were generated.

### Functional annotation

Gene functions of all the assembled unigenes were annotated to NCBI non-redundant protein sequences (Nr, http://www.ncbi.nlm.nih.gov/protein/), Kyoto Encyclopedia of Genes and Genomes (KEGG, http://www.kegg.jp/), Gene Ontology (GO, http://www.geneontology.org/), evolutionary genealogy of genes: Non-supervised Orthologous Groups (eggNOG, http://eggnog.embl.de/version_4.0.beta/) and Swiss-Prot (http://web.expasy.org/docs/swiss-prot_guideline.html).

### Quantification of gene expression levels and functional enrichment

Clean reads were mapped to assembled transcriptome using RSEM to obtain the read count for each gene and the expression level of each gene was estimated using fragments per kilobase of transcript sequence per millions base pairs sequenced (FPKM). Differentially expressed genes (DEGs) were identified with a standard of fold change and p-value (|log_2_ (fold change)| > 1 and p-value< 0.05).

After obtaining the DEGs, The function was predicted by GO analysis in three categories: cellular component (CC), molecular function (MF) and biological process (BP). KEGG was performed to identify the cellular signal pathways that DEGs involved in. The threshold of significance for GO and KEGG analyses was defined by p value < 0.05.

### Simple Sequence Repeat (SSR) identification

MISA(Version 1.0, http://pgrc.ipkgatersleben.de/misa/misa.html) was used to identify SSR motifs. For di-, tri-, tetra-, penta- and hexa-nucleotides, the lowest number of base repeats was 10, 6, 5, 5, 5 and 5, respectively. In addition, if the distance between two SSRs was less than 100 bp, they would be merged into a composite SSR.

### Quantitative real-time PCR (qRT-PCR)

In order to validate the RNA-seq results, eight differentially expressed genes between ovary and testis were chosen for qRT-PCR analysis with the same RNA samples from Illumina sequencing. Gene-specific primers were designed using Primer Premier5.0. B2M was the internal reference gene. First strand cDNA was synthesized using the RevertAid First Strand cDNA synthesis kit (Thermo Fisher Scientific, MA, USA). qRT-PCR was carried out with an ABI Q6 real-time PCR machine (Applied Biosystem Inc., MA, USA) using QuantiFast SYBR Green PCR Kit (Qiagen, Hilden, Germany) in 10 μL reaction mixture with 1μL cDNA as template. The PCR cycling parameters were: 95°C for 10 min; 45 cycles of 95°C for 15 s and 60°C for 60 s followed by a melting curve. Every sample was performed in triplicate. Amplicons were verified through melting-curve analysis. The 2-ΔΔCT method was used to determine the relative gene expression. Quantitative data were expressed as means±SD (standard deviation) representing the relative expression. The significance of differences in expression was determined using one-way ANOVA followed by t-test with a threshold significance level of P<0.05. The primers were displayed in S1 Table.

### Investigation of SSR polymorphism

A total of 10 primer pairs (S2 Table) were synthesized and 210 individuals of *C*. *lucidus*s from 7 different locations, including Lianyungang (LYG), Zhoushan (ZS), Chongming (CM), Wenzhou (WZ), Xiamen (XM), Ningde (ND), Dafeng (DF), were selected for polymorphism investigation with the SSRs. Total DNA was extracted from dorsal muscles using traditional phenol-chloroform extraction protocols as described by Ma et al. [17]. The total volume of PCR reactions were 20 μL containing 1 μL template DNA, 1μL forward and reverse primers (10 μM), 1 μL 2.5 mM dNTPs, 1 μL EasyTaq DNA polymerase (Beijing Trans Gen Biotech Co., Ltd. China), 2 μL 10×EasyTaq buffer and 13 μL ddH2O. Amplification program of PCR was as follows: denaturation for 5 min at 94°C, 35 cycles of 94°C for 30 s, 59°C for 30 s, and 72°C for 1min, and a final step at 72°C for 10 min. Alleles were detected on a 12% polyacrylamide gel. UPMGA evolution tree was constructed using MEGA 5.1 based on Nei genetic distance.

### Statistical analysis

Statistical analysis was performed through the SPSS 13.0 statistical software (Chicago, IL, USA). The data were expressed as mean±standard deviation (SD). Comparison between two difference independent groups was performed by two-sided Student’s test. A p-value < 0.05 was considered to be a statistically significant difference.

## Results

### Sequencing and *de novo* assembly

Totally, the transcriptome sequencing generated 237,519,998 raw reads (60,954,060, 57,468,802, 61,486,012 and 57,611,124 reads from brain, liver, ovary and testis tissues of *C*. *lucidus*, respectively). After removing low quantity reads and trimming adaptor reads, 60,322,004 (98.96%), 57,044,284 (99.26%), 60,867,978 (98.99%) and 57,087,688 (99.09%) clean reads from brain, liver, ovary and testis tissues of *C*. *lucidus* were obtained, respectively (S3 Table). *De novo* assembly of clean reads generated a total of 189,181 transcripts with an average length of 818 bp and a N50 length of 1463 bp. The longest transcript of each gene was selected and yielded 131,168 unigenes. The average length of unigenes was 644 bp and the N50 length was 1033 bp (S4 Table).

### Functional annotation

**Fig 1:**
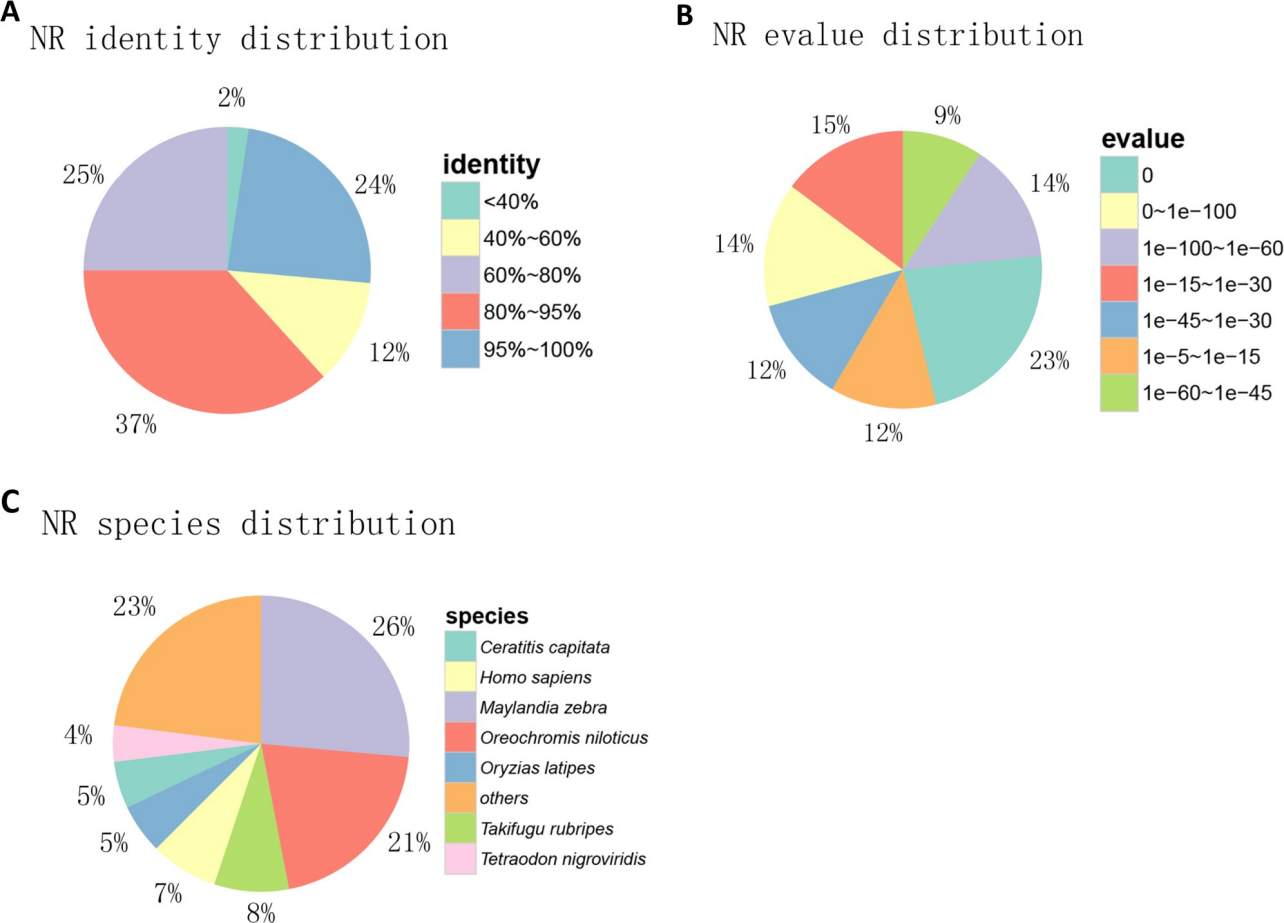
Transcripts BLAST against NR. (A) The similarity of mapped sequences with genes in NR database. (B) E-value distribution. (C) Species distribution.

Unigenes were annotated using NR, GO, KEGG and Swissport databases, and a 10^−5^ e-value cut-off value was used. 51,590 (39.33%), 27,708 (21.12%), 12,883 (9.82%) and 44,051 (33.58%) significant hits were produced, respectively. Among these unigenes, 53,200 (40.56%) were annotated at least in one database and 8851 (6.75%) unigenes were annotated in all four databases (S5 Table). The similarity distribution of the top hits showed that 61% of the mapped sequences had similarities more than 80%, while 37% of the hits had similarities ranging from 40% to 80% (Fig 1A). The E-value distribution had a comparable pattern with 51% of the mapped sequences with high homologies (<1e-60), whereas 48% of the homological sequences ranged between 1e-5 to 1e-60 (Fig 1B). The species distribution of NR BLAST matches showed that the top two species was *Maylandia zebra* (26%) and *Oreochromis niloticus* (21%) (Fig 1C).

### GO and KEGG analysis of *C*. *lucidus* transcriptomes

**Fig 2:**
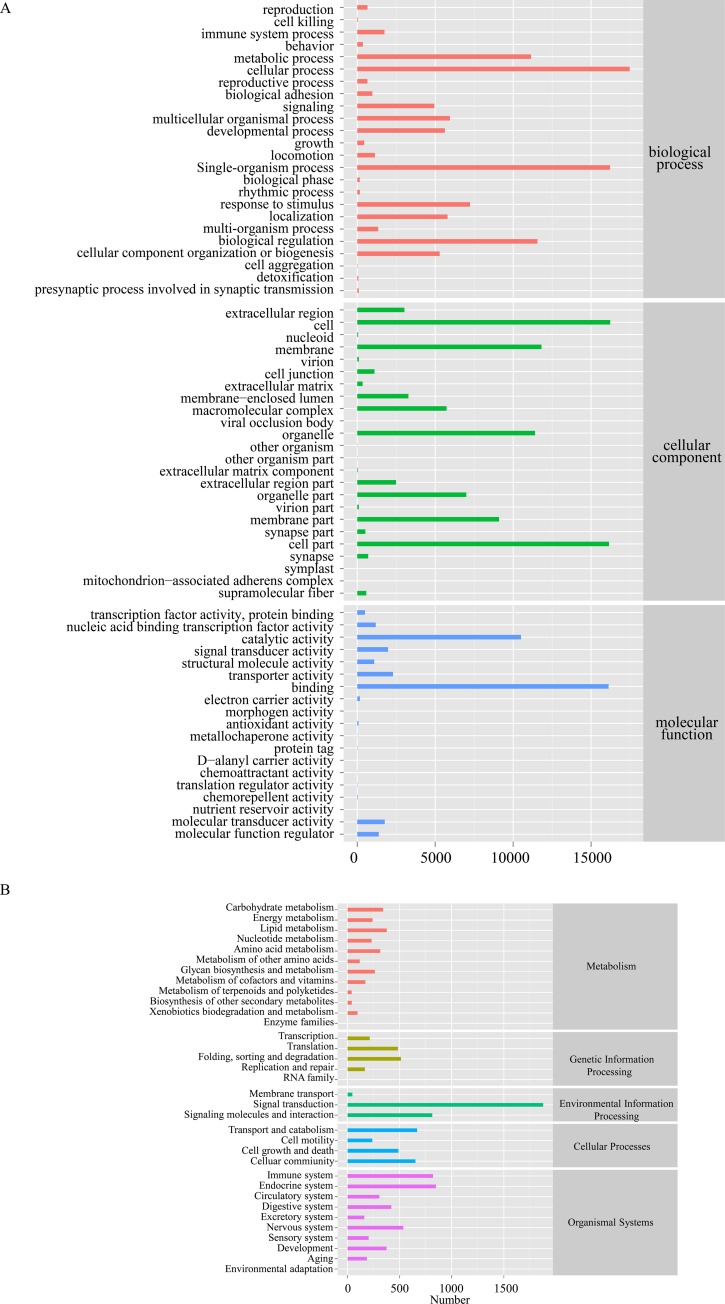
Functional analysis of *C*. *lucidus* unigenes. (A) GO annotation of unigenes from *C*. *lucidus* based on biological processes, molecular function, and cellular components. (B) KEGG annotation of unigenes based on metabolism, genetic information processing, environmental information processing, cellular processes, and organismal systems.

A total of 27,708 unigenes were clustered into 67 GO terms under three different GO categories containing (biological process (BP), cellular component (CC) and molecular function (MF). The number of unigenes in each GO term were shown in Fig 2A. The top 3 terms ranked by number of unigenes in BP were cellular process (17,477), single-organismal process (16,219) and biological regulation (11,558). For CC, unigenes were mainly involved in cell (16,229), cell part (16,151) and membrane (11,815). For MF, binding (16,123) was the most prevalent, followed by nucleic acid binding catalytic activity (10,504) and signal transducer activity (1,974).

To further explore the biological pathways, all unigenes were mapped in KEGG database and 12,883 unigenes were mapped to 32 categories, among which signal transduction (1,877), endocrine system (849), immune system (820), signaling molecules and interaction (814) and transport and catabolism (668) were the largest five groups (Fig 2B).

### Analysis of differentially expressed unigenes

**Fig 3:**
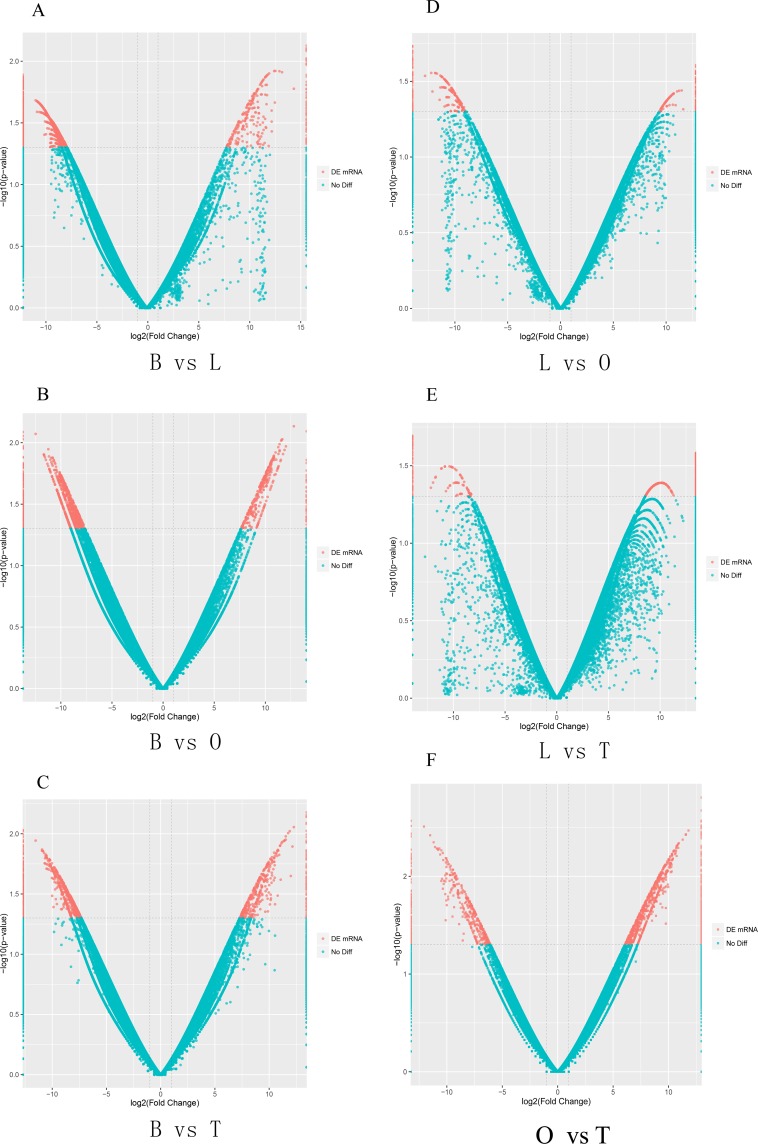
Analysis of differentially expressed unigenes. Volcano plot shows differentially expressed unigenes between each pair of brain (B), liver (L), ovary (O) and testis (T) tissues. (A) Differentially expressed unigenes in B vs L. (B) Differentially expressed unigenes in B vs O. (C) Differentially expressed unigenes in B vs T. (D) Differentially expressed unigenes in L vs O. (E) Differentially expressed unigenes in L vs T. (F) Differentially expressed unigenes in O vs T. Red dots represent differentially expressed unigenes. Blue dots represent not-significant differentially expressed unigenes. The red dots on the left represented the up-regulated unigene expression of the samples on the left, and the red far dots on the right represented the up-regulated unigene expression of the sample on the right.

**Fig 4:**
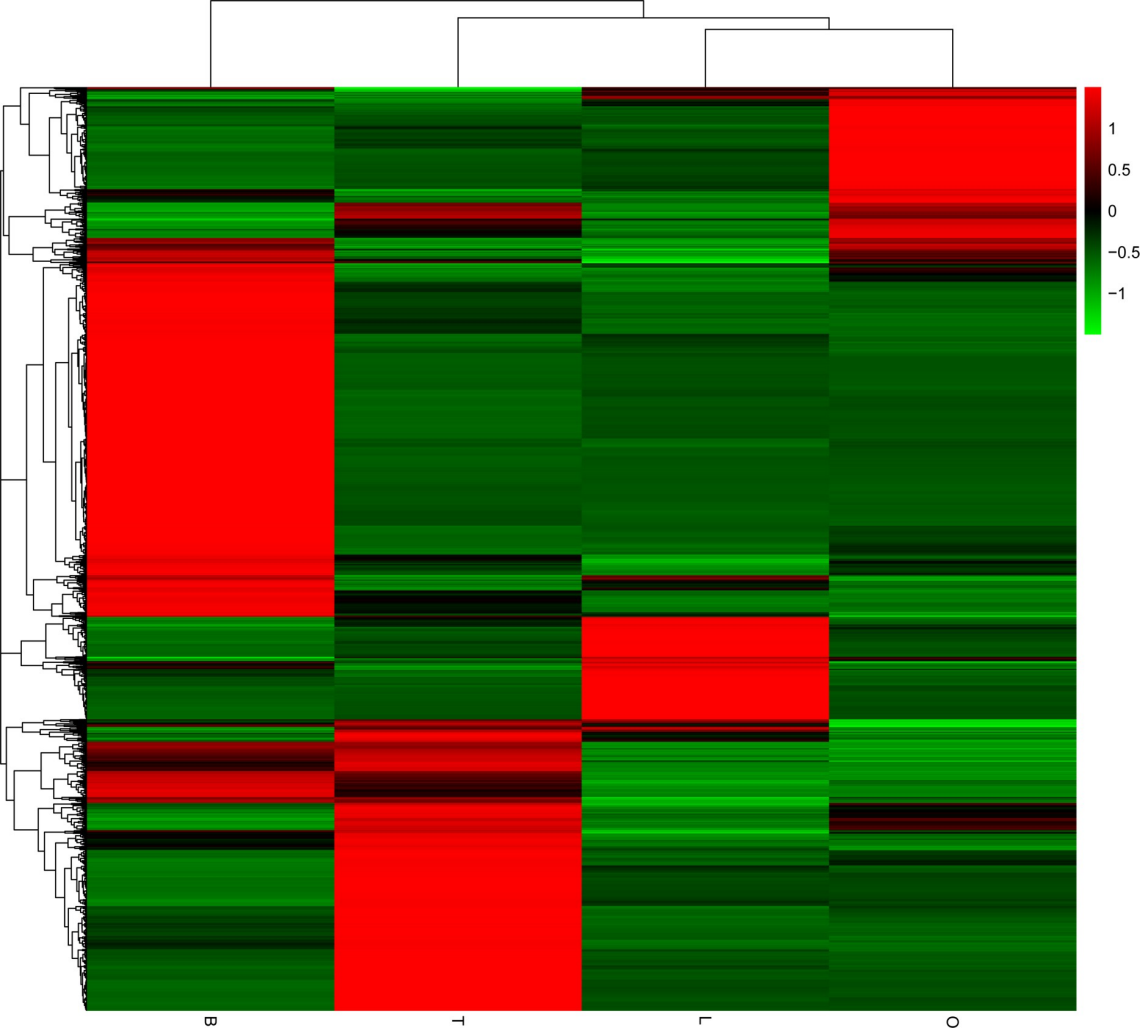
Cluster heatmap of unigenes between any two tissues of B (brain), L (liver), O (ovary) and T (testis). Different rows represent different unigenes. Different colors represent different cluster grouping information, red represents unigenes with high expression, and green represents unigenes with low expression.

In order to search for genes involved in reproduction, we identified differentially expressed unigenes among brain, liver, testis and ovary tissues. Between brain and liver, 992 genes were upregulated in brain and 264 genes were upregulated in liver. Between brain and ovary, 749 genes were upregulated in brain and 317 genes were upregulated in ovary. Between brain and testis, 685 genes were upregulated in brain and 388 genes were upregulated in testis. Between liver and ovary, 211 genes were upregulated in liver and 114 genes were upregulated in ovary. Between liver and testis, 138 genes were upregulated in liver and 315 genes were upregulated in testis. Between ovary and testis, 442 genes were upregulated in ovary and 846 genes were upregulated in testis (Fig 3, S6 Table). Additionally, cluster heatmap of unigenes revealed that more unigenes were highly expressed in brain, while less unigenes were highly express in liver. A gene was identified as organ-specific when the FPKM was at least 10 in one organ whereas less than 1 in other organs. By this standard, 2,143, 304, 333, 556 unigenes were found specifically expressing in brain, liver, ovary and testis, respectively (Fig 4).

### Functional analysis of differentially expressed unigenes

**Fig 5:**
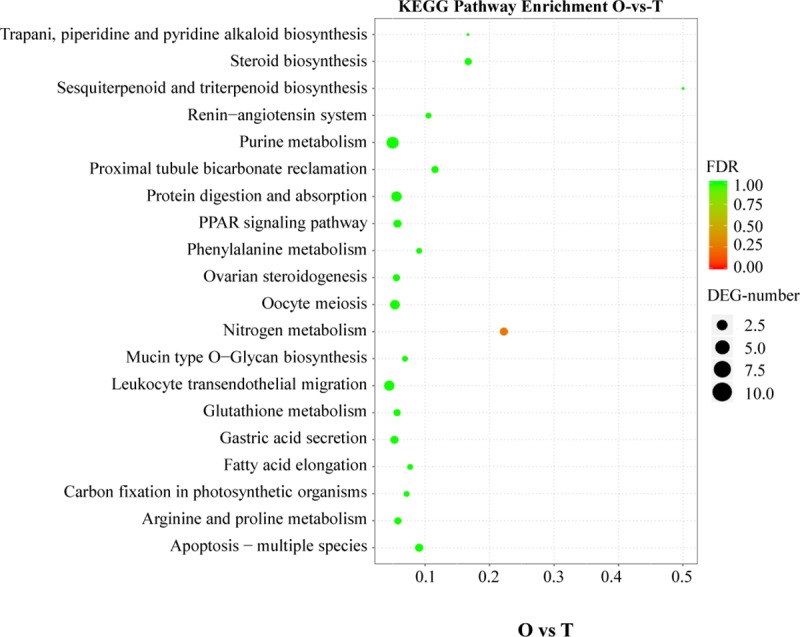
KEGG pathway enrichment analysis of differently expressed genes between ovary (O) and testis (T) tissues. The size of the dot indicates how much the differentially expressed genes are annotated into the pathway, and the color indicates the significance of the pathway. The figure shows the 20 most significant pathways between O and T tissues.

To investigate pathways connected to reproduction and gonad development, we particularly focused on differentially expressed unigenes between ovary and testis. These differentially expressed unigenes were assigned to 165 pathways. Differentially expressed unigenes between ovary and testis were mainly associated with PPAR signaling pathway, ovarian steroidogenesis, oocyte meiosis, steroid hormone biosynthesis, apoptosis-multiple species and proximal tubule bicarbonate reclamation (Fig 5).

### Identification and characterization of genes involved in reproduction and sexual development

Based on the *de novo* assembly and annotation, 652 and 650 candidate unigenes were identified for reproduction (GO: 0000003) and reproductive process (GO: 0022414) respectively. Furthermore, 8 unigenes were enriched in both terms of reproduction, sex differentiation and development, implying that they dominated the regulation of reproduction and sex development. Among the 8 unigenes, *HYAL* (hyaluronidase), *KLHL10* (Kelch-like protein 10), *ROPN1L* (rhophilin associated tail protein 1 like), *ODF3L2* (outer dense fiber of sperm tails 3 like 2), *SYCP3* (synaptonemal complex protein 3) and *SRPK3-like* (serine/threonine-protein kinase 3-like) were upregulated in testis, whereas, *BMP15* (bone morphogenetic protein 15) and *RGS14* (regulator of G protein signaling 14) were upregulated in ovary (Table 1).

**Table 1:** Different genes in GO terms related to reproduction.

ID	O	T	foldChange(T/O)	log_2_FoldChange	pvalue
**c35502_g1(HYAL)**	0	1950	-	-	0.003
**c55252_g1(KLHL10)**	2	1765	882.5	9.79	0.008
**c55445_g1(BMP15)**	9245	20	0.002	-8.85	0.015
**c28789_g1(ROPN1L)**	15	4292	286.13	8.16	0.019
**c47852_g1(ODF3L2)**	0	217	-	-	0.023
**c65782_g1(SYCP3)**	53	20287	382.77	8.58	0.024
**c66108_g1(RGS14)**	19878	48	0.003	-8.18	0.026
**c54644_g1(SRPK3-like)**	0	157	-	-	0.028

### Identification of SSRs and SSR marker polymorphism

SSRs were predicted using MISA 1.0 (http://pgrc.ipkgatersleben.de/misa/misa.html). Totally, we found 35,476 SSRs. Among them, the most affluent repeat motif was mono- and di-nucleotide repeats, accounting for 41.18% (14,608) and 38.23% (13,563), respectively, followed by tri- (18.74%, 6,648), tetra- (1.73%, 614), penta- (0.09%, 31) and hexa-nucleotide (0.03%, 12) (S7 Table).

To study the genetic structure of different geographic populations of *C*. *lucidus*, 10 primer pairs were designed to validate the amplification and polymorphism in *C*. *lucidus* collected from 7 different regions. Among these primer pairs, one pair (primer 187) exhibited polymorphisms among the seven colonial *C*. *lucidus* (S1A, S1B Fig). The polymorphic SSR marker was then used to perform genetic correlation analysis among the *C*. *lucidus* groups. The UPGMA clustering produced a dendrogram that separated the *C*. *lucidus* into two main groups, from which Ningde (ND) completely separate from the others. Moreover, Lianyungang (LYG) and Zhoushan (ZS) formed a sub-cluster, while Chongming (CM) and Dafeng (DF) formed a sub-cluster (S1C Fig).

### Validation of gene expression using qRT-PCR

**Fig 6:**
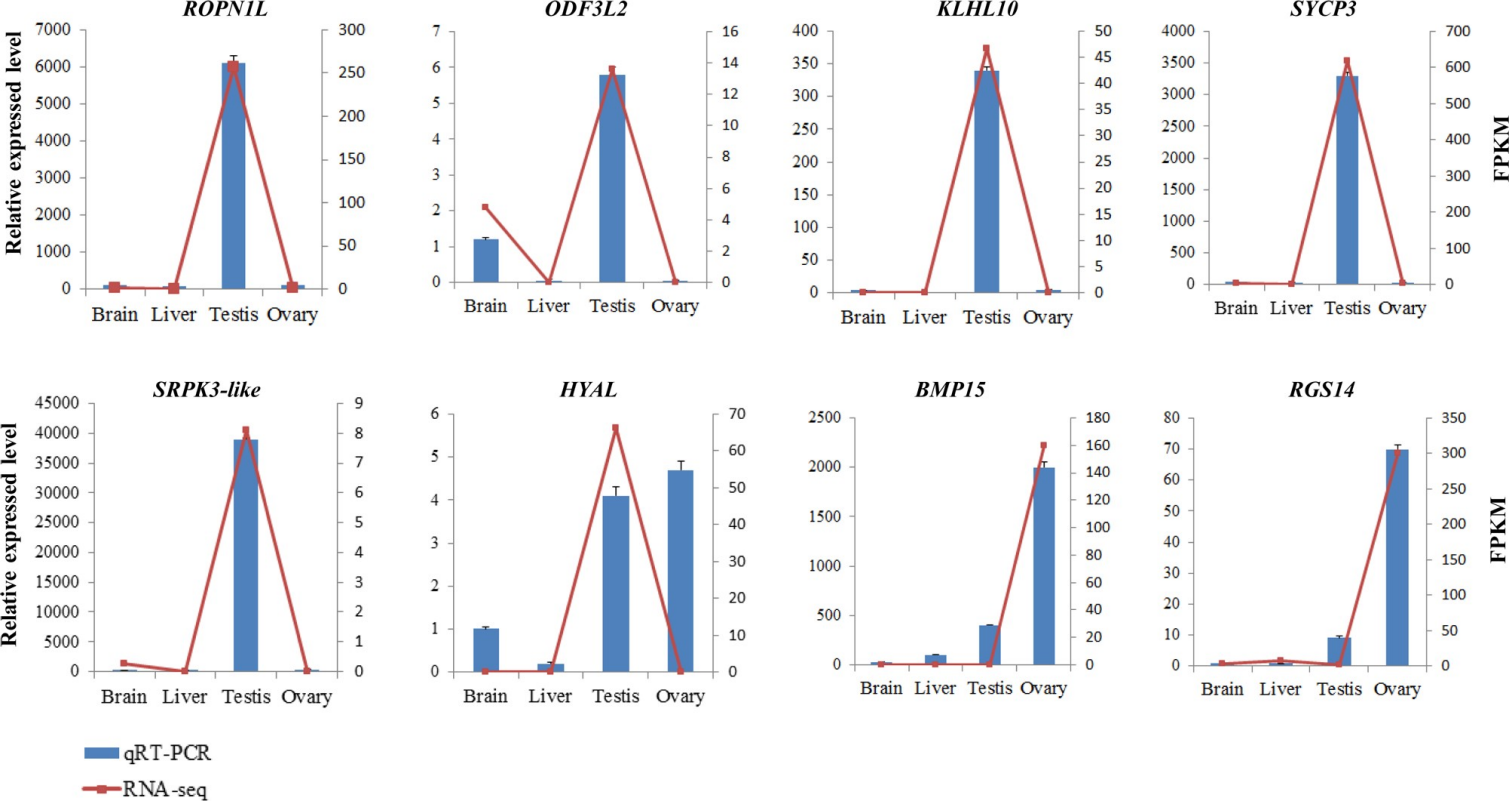
Genes involved in the reproduction were verified by qRT-PCR. *ROPN1L*: rhophilin associated tail protein 1 like, *ODF3L2*: outer dense fiber of sperm tails 3 like 2, *KLHL10*: Kelch-like protein 10, *SYCP3*: synaptonemal complex protein 3, *SRPK3-like*: serine/threonine-protein kinase 3-like, *HYAL*: hyaluronidase, *BMP15*: bone morphogenetic protein, *RGS14*: regulator of G protein signaling 14. * represents significant difference between the two groups (P<0.05).

To verify the expression of genes involved in reproduction, 8 genes enriched in reproduction terms were validated by qRT-PCR analysis. In accordance with the results of RNA-seq, qRT-PCR showed that *ROPN1L*, *KLHL10*, *ODF3L2*, *SYCP3* and *SRPK3-like* were specific expressed in testis, whereas BMP15 and RGS14 were specific expressed in ovary (Fig 6).

## Discussion

Researches have reported that *de novo* transcriptome sequencing has been used in many aquatic animals [21]. Meng et al. obtained 128,904,126 clean reads in all from the ovary and testis tissues of *Portunus trituberculatus* by RNA-Seq [22]. Besides, gonadal transcriptomic sequencing of *Pelteobagrus fulvidraco* generated 193,477,064 clean reads by Lu et al. [23]. However, little is known about the transcriptome of *C*. *lucidus* organs. In our study, we gathered 60,322,004 (98.96%), 57,044,284 (99.26%), 60,867,978 (98.99%) and 57,087,688 (99.09%) paired-end clean reads from brain, liver, ovary and testis tissues of *C*. *lucidus*, assembled 189,181 transcripts and identified 131,168 unigenes. It can provide more genetic resources for the research of reproduction in *C*. *lucidus*.

HPG axis is the sex endocrine system of fish and plays an important role in fish reproduction via a cascade of hormones [24]. Transcriptome analysis of HPG axis from *Lateolabrax maculatus* indicated 748, 349 and 319 unigenes were uniquely expressed in the brain, ovary and testis tissues, respectively, while 26623 unigenes were commonly expressed in all HPG tissues [25]. Xu et al. analyzed the gene expression of HPG axis in *Cyprinus carpio* and suggested that 9, 28, and 212 genes were differentially expressed in hypothalamus, pituitary, and ovary, respectively [26]. Therefore, gene expression of the HPG axis was different in different kinds of fish. In this study, the vast majority of the organ-specific expression was found in brain, containing 2,143 unigenes, whereas, only 304, 333 and 556 in liver, ovary and testis of *C*. *lucidus*, respectively. In addition, 1288 genes were differentially expressed between ovary and testis. Studies of the expression of genes associated with the HPG axis can promote understanding the role of HPG axis in the regulation of fish reproduction [27]. Wang et al. found that altered expressions of genes in the HPG axis was closely related to the disrupted oogenesis in yellow catfish [28]. Therefore, the expression of genes associated with the HPG axis in the ovary and testis might be altered, resulting in the regulation of the sex determination and reproduction in *C*. *lucidus*. These data in our study will provide a foundation for futher understanding the molecular regulation of gonadal development and reproduction.

GO and KEGG analyses were used for functional categorization of the assembled unigenes, and for auquiring information of gene functions that can contribute to predicting the role of protein interaction networks in cells [29]. In our study, cellular process, cell, binding were the most abundant classifications in biological process, cellular component and molecular function, respectively. This result was similar to the result of Meng et al. who performed *de novo* transcriptome sequencing of *Portunus trituberculatus* ovary and testis [22]. The KEGG annotation indicated signal transduction was the largest group that a lot of genes and pathway might be involved. Signal transduction is the process by which physical and chemical signals are transmitted through cells, and this process is crucial for the development and maintenance of normal gametogenesis [30]. In addition, in order to identify pathways participating in reproduction, we carried out KEGG analysis of differentially expressed unigenes between ovary and testis and found a large number of genes which were mainly associated with PPAR signaling pathway, ovarian steroidogenesis, oocyte meiosis, steroid hormone biosynthesis, apoptosis-multiple species and proximal tubule bicarbonate reclamation. Therefore, it can help us further understanding the molecular mechanism of reproduction in *C*. *lucidus*.

Hyaluronidase was an important component of fertilization in mammals for acrosomal reaction [31]. In this study, *HYAL*, the gene encoding hyaluronidase, was exactly upregulated in testis. It indicated that hyaluronidase may contribute to fertilization in *C*. *lucidus*. Besides, SYCP3, the gene encoding synaptonemal complex protein 3, which closely connect to synapsis in meiosis [32]. In the present study, *SYCP3* was upregulated in testis. In agreement with our result, this gene was examined to reflect the level of meiosis and spermatogenesis in zebrafish, tilapia and catfish [33–35]. In addition, *BMP15* belongs to the subgroup of transforming growth factor β (TGFβ) superfamily, and plays important roles in regulating ovarian functions [36]. Dranow et al. found *BMP15* was required for maintaining female sexual fate in zebrafish [37]. In our qRT-PCR, *BMP15* was highly expressed in ovary, suggesting that it might participate in sex determination.

SSRs is one of the most widely used DNA molecular markers [38]. SSR markers are increasingly used as marker systems in molecular genetics studies because of their rich polymorphism, high high conservation, and reproducibility [39, 40]. For the conservation of *C*. *lucidus*, SSRs can act as effective molecular markers for quantifying genetic diversity. In our study, we screened 35,476 SSRs. The mononucleotide repeats (14608, 41.18%) were predominant followed by dinucleotide (13563, 38.23%) and tricleotide repeats (6648, 18.74%). This was similar to the reported results of Yue et al. who identified 12151 SSRs in which mononucleotide repeat motifs accounted for the first largest group in the *Acipenser sinensis* transcriptome [41]. Moreover, we performed genetic correlation analysis among the *C*. *lucidus* groups collected from 7 different regions using polymorphic SSR markers and discovered Lianyungang (LYG) and Zhoushan (ZS) formed a sub- cluster, Chongming (CM) and Dafeng (DF) formed a sub-cluster, while Ningde (ND) completely separate from the others. It indicated that different regional population of *C*. *lucidus* had different genetic relationships. It provides a basis for further study on the genetic structure of different geographic populations of *C*. *lucidus*.

## Conclusion

Our *de novo* transcriptome sequencing generated 131,168 unigenes in all, among which a large number of genes were potentially involved in reproduction and sex determination. This study will enrich genetic information for *C*. *lucidus* and provide a basis for further research on the molecular regulation of reproduction and sex determination of this species. Additionally, 35,476 SSRs were identified, which will serve as molecular markers for the research of genetic diversity in *C*. *lucidus*.

## Supporting information

S1 Table PCR primers for the validation of RNA-seq data by qRT-PCR. (DOCX)

S2 Table S2 Table 2 Primers used for SSR polymorphic analysis in this study. (DOCX)

S3 Table Data analysis of raw reads and clean reads in *C*. *lucidus* from B (brain), L (liver), O (ovary) and T (testis). (DOCX)

S4 Table Statistics of the transcriptome sequencing and *de novo* assembly data in *C*. *lucidus*. (DOCX)

S5 Table Summary of function annotations of unigenes in *C*. *lucidus*. (DOCX)

S6 Table Unigenes differentially expressed in each two organs. (DOCX)

S7 Table SSRs identified in *C*. *Lucidus*. (DOCX)

S1 Fig SSR polymorphism analysis of seven *C*. *lucidus* populations from different locations. (A and B) Polyacrylamide gel electrophoresis shows the SSR polyacrylamide. (C) Dendrogram constructed with UPGMA clustering of C. Lucidus from seven different locations. LYG: Lianyungang, ZS: Zhoushan, CM: Chongming; WZ: Wenzhou, XM: Xiamen, ND: Ningde, DF: Dafeng.
(TIF)
