# Supplementary material for: De novo transcriptome assembly of four organs of Collichthys lucidus and identification of genes involved in sex determination and reproduction
Source: PLoS One. 2020 Mar 27;15(3):e0230580. doi: 10.1371/journal.pone.0230580 (PMC7100973; doi:10.1371/journal.pone.0230580)
Supplement: S1 Table — (DOCX) [file pone.0230580.s001.docx]

**Table S1 PCR primers for the validation of RNA-Seq data by qRT-PCR.**

| ID | Forward Primer Sequence 5'-3' | Reverse Primer Sequence 5'-3' |
| --- | --- | --- |
| c35502_g1  (HVAL) | 5' CCAGAGCATCGCCATATTCTACACAG 3' | 5' TCCACTCCTCAAAGTCAAGCAC 3' |
| c55252_g1  (KLHL10) | 5' CTTGCACATGGCCGTTGAAG 3' | 5' GCGATAAGCACGAGGAGATT 3' |
| c55445_g1  (BMP15) | 5' AGCCTCGTTCATCCACCTCC 3' | 5' GCTGCCGTTCTCTACAGG 3' |
| c28789_g1 (ROPN1L) | 5' GACCAGCTTGAGTCCCTG 3' | 5' TGCAGGCTGCTGAGGAAC 3' |
| c47852_g1  (ODF3L2) | 5' CGAATGAAAGCAGAGAAGG 3' | 5' GAGGGCCCATTAGCGGAG 3' |
| c65782_g1  (SYCP3) | 5' CCAGCCAAGAAGAGACCTG 3' | 5' TTCAGACTCCCACTGCTGC 3' |
| c66108_g1  (RGS14) | 5' GCCAAGACCGAGGAGAAGGACCT 3' | 5' TGGCAAGCTGTGGAAGTCTG 3' |
| c54644_g1  (SRPK3-like) | 5' ACGGACATCAAGCCAGAG 3' | 5' ATGCGAAGGAGGAAGTCTG 3' |
