# Supplementary material for: De novo transcriptome assembly of four organs of Collichthys lucidus and identification of genes involved in sex determination and reproduction
Source: PLoS One. 2020 Mar 27;15(3):e0230580. doi: 10.1371/journal.pone.0230580 (PMC7100973; doi:10.1371/journal.pone.0230580)
Supplement: S2 Table — (DOCX) [file pone.0230580.s002.docx]

**Table S2 Primers used for SSR polymorphic analysis in this study**

| ID | Primers name | Primers sequence 5'-3' | size |
| --- | --- | --- | --- |
| c100190_g1 | 190F | GGGGGAAGAAGAGAAAGTGG | 20 |
|  | 190R | GACACAGAGGGCAGAGCAC | 19 |
| c80490_g1 | 490F | AGCTAGCGCTCAATGGAAAG | 20 |
|  | 490R | CACAAATATGCAGCAGTCGG | 20 |
| c99257_g1 | 257F | TCCTGACCGACTAGGCTGAT | 20 |
|  | 257R | GCTGATGTGTGTGTGTCCGT | 20 |
| c40180_g1 | 180F | AGCTCGCTCTCTCTCTCCCT | 20 |
|  | 180R | GGGAGTTTTTGTCCCCAGTT | 20 |
| c8187_g1 | 187F | TCCATTAGGGAAACTGGTGC | 20 |
|  | 187R | TGCACACAAGCATGCATAAA | 20 |
| c101052_g1 | 052F | CACACATCTTTTCCAAAATGC | 21 |
|  | 052R | GAAACAGAAAAGCGAGTGGG | 20 |
| c68969_g1 | 969F | ACCGTGAAGAGTCACCATCC | 20 |
|  | 969R | ATATATCTGTGCGGGCTGCT | 20 |
| c89801_g1 | 801F | GTCCCGTTCTCGATCCCT | 18 |
|  | 801R | GAGACAGACAGAGAGACAGACCAG | 24 |
| c105626_g1 | 526F | CGCCGAGTACGTAACCCATA | 20 |
|  | 526R | GAATCAATGGGGATGTGGTC | 20 |
| c26936_g1 | 936F | TAATGGATGGGGCAGAGAAG | 20 |
|  | 936R | TAGACTGCACGAGCATACCG | 20 |
