# Supplementary material for: De novo transcriptome assembly of four organs of Collichthys lucidus and identification of genes involved in sex determination and reproduction
Source: PLoS One. 2020 Mar 27;15(3):e0230580. doi: 10.1371/journal.pone.0230580 (PMC7100973; doi:10.1371/journal.pone.0230580)
Supplement: S3 Table — (DOCX) [file pone.0230580.s003.docx]

**Table S3 Data analysis of raw reads and clean reads in *C. lucidus* from brain, liver, ovary and testis tissues**

| Sample | Raw reads number | Clean reads number | Clean reads (%) |
| --- | --- | --- | --- |
| B | 60,954,060 | 60,322,004 | 98.96 |
| L | 57,468,802 | 57,044,284 | 99.26 |
| O | 61,486,012 | 60,867,978 | 98.99 |
| T  Total | 57,611,124  237,519,998 | 57,087,688  235,321,954 | 99.09  - |
