# Supplementary material for: De novo transcriptome assembly of four organs of Collichthys lucidus and identification of genes involved in sex determination and reproduction
Source: PLoS One. 2020 Mar 27;15(3):e0230580. doi: 10.1371/journal.pone.0230580 (PMC7100973; doi:10.1371/journal.pone.0230580)
Supplement: S4 Table — (DOCX) [file pone.0230580.s004.docx]

**Table S4 Statistics of the transcriptome sequencing and de novo assembly data in** ***C. lucidus***

| Type | Transcripts | Unigenes |
| --- | --- | --- |
| Total sequence number | 189181 | 131168 |
| Total length (bp) | 154673659 | 84460085 |
| GC (%) | 51.03 | 49.88 |
| Max length (bp) | 26592 | 26592 |
| Average length (bp) | 818 | 644 |
| N50 (bp) | 1463 | 1033 |
| N90 (bp) | 307 | 263 |
