# Supplementary material for: De novo transcriptome assembly of four organs of Collichthys lucidus and identification of genes involved in sex determination and reproduction
Source: PLoS One. 2020 Mar 27;15(3):e0230580. doi: 10.1371/journal.pone.0230580 (PMC7100973; doi:10.1371/journal.pone.0230580)
Supplement: S5 Table — (DOCX) [file pone.0230580.s005.docx]

**Table S5 Summary of function annotations of unigenes in *C. lucidus***

| Datebase | Number of annotated unigene | Percentage of  annotated unigene |
| --- | --- | --- |
| GO | 27708 | 21.12% |
| KEGG | 12883 | 9.82% |
| NR | 51590 | 39.33% |
| SWISSPROT | 44051 | 33.58% |
| All annotated transcripts | 53200 | 40.56% |
| Transcripts identified in all four databases | 8851 | 6.75% |
