# Supplementary material for: De novo transcriptome assembly of four organs of Collichthys lucidus and identification of genes involved in sex determination and reproduction
Source: PLoS One. 2020 Mar 27;15(3):e0230580. doi: 10.1371/journal.pone.0230580 (PMC7100973; doi:10.1371/journal.pone.0230580)
Supplement: S6 Table — (DOCX) [file pone.0230580.s006.docx]

**Table S6 Unigenes differentially expressed in each two organs**

| Case | control | Up-regulateed unigenes | Down-regulated unigenes | Total DE unigenes |
| --- | --- | --- | --- | --- |
| B | L | 992 | 264 | 1256 |
| B | O | 749 | 317 | 1066 |
| B | T | 685 | 388 | 1073 |
| L | O | 211 | 114 | 325 |
| L | T | 138 | 315 | 453 |
| O | T | 442 | 846 | 1288 |
