# Supplementary material for: De novo transcriptome assembly of four organs of Collichthys lucidus and identification of genes involved in sex determination and reproduction
Source: PLoS One. 2020 Mar 27;15(3):e0230580. doi: 10.1371/journal.pone.0230580 (PMC7100973; doi:10.1371/journal.pone.0230580)
Supplement: S7 Table — (DOCX) [file pone.0230580.s007.docx]

**Tbale S7 SSRs identified in *Collichthys. Lucidus***

| Item | Number |
| --- | --- |
| Total number of SSRs | 34,476 |
| Number of mono-nucleotide repeats | 14,608 |
| Number of di-nucleotide repeats | 13,563 |
| Number of tri-nucleotide repeats | 6,648 |
| Number of tetra-nucleotide repeats | 614 |
| Number of penta-nucleotide repeats | 31 |
| Number of hexa-nucleotide repeats | 12 |
