# Supplementary figures and images for: De novo transcriptome assembly of four organs of Collichthys lucidus and identification of genes involved in sex determination and reproduction
Source: PLoS One. 2020 Mar 27;15(3):e0230580. doi: 10.1371/journal.pone.0230580 (PMC7100973; doi:10.1371/journal.pone.0230580)

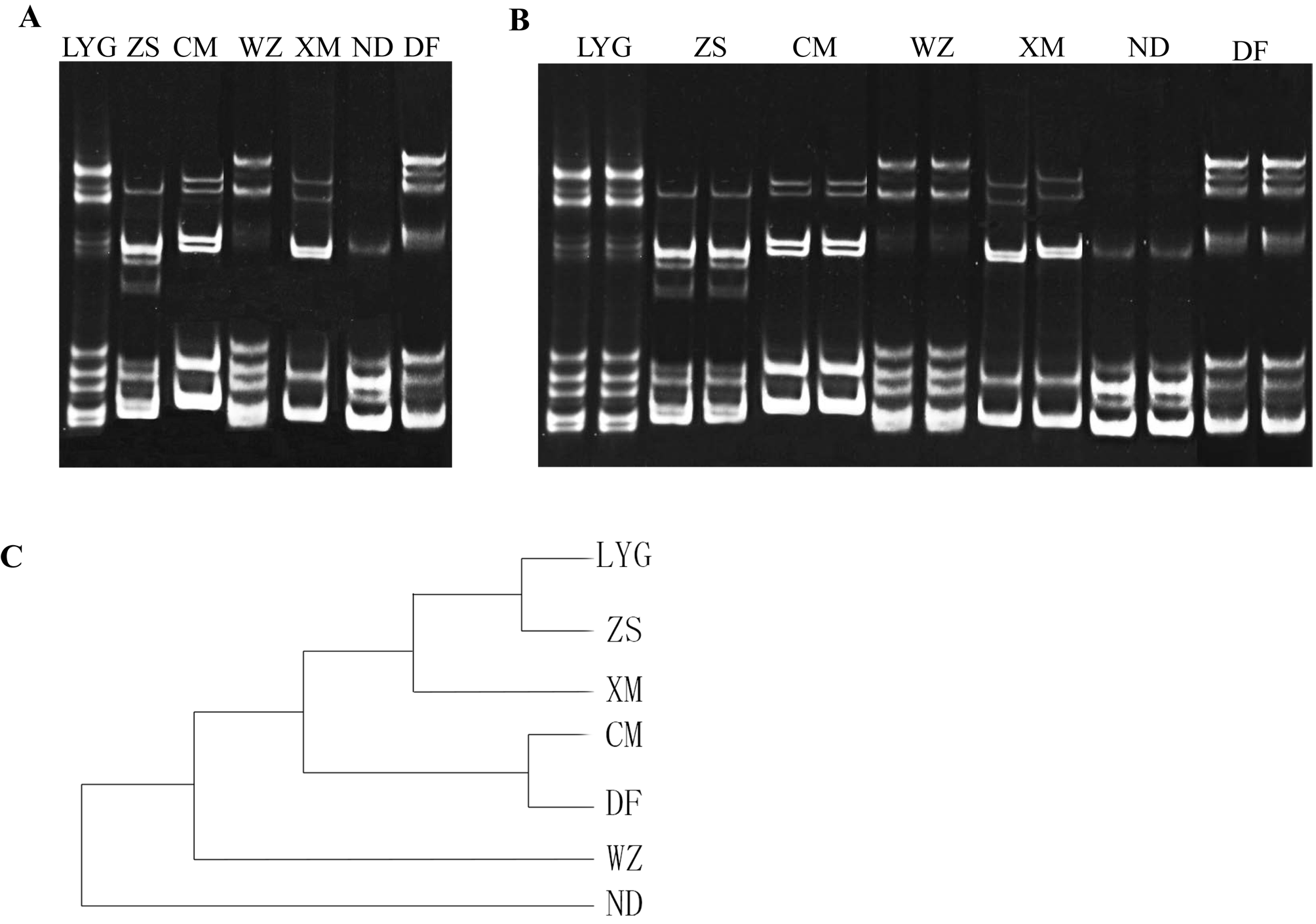

Supplement: S1 Fig — (A and B) Polyacrylamide gel electrophoresis shows the SSR polyacrylamide. (C) Dendrogram constructed with UPGMA clustering of C. Lucidus from seven different locations. LYG: Lianyungang, ZS: Zhoushan, CM: Chongming; WZ: Wenzhou, XM: Xiamen, ND: Ningde, DF: Dafeng. (TIF) [file pone.0230580.s008.tif]
